# Supplementary material for: Discharge against medical advice in Special Care Newborn Unit in Chattogram, Bangladesh: Prevalence, causes and predictors
Source: PLoS One. 2023 Apr 20;18(4):e0284705. doi: 10.1371/journal.pone.0284705 (PMC10118186; doi:10.1371/journal.pone.0284705)
Supplement: Attachment — Submitted filename: Rebuttal letter.pdf [file pone.0284705.s002.pdf]

Detailed Response to Reviewers

Reviewer 1

Comment 1: Data seemed to be more than 5 years old which make the findings less relevant.

Response: I would like to thank you very much to reviewer 1 for reviewing the manuscript and providing your expert comments and opinions on the manuscript. All the comments were welcomed and carefully studied. We apologize for our late publication. Actually we discussed the study findings with our hospital authority. On the basis of these findings, a new kangaroo-mother-care unit was established and daily counseling by consultants with the mother and attendants were started. It is expected that this sort of study will always help to undertake similar relevant steps for better patient management and outcome.

Comment 2: Sample size has not been calculated

Response: We included all the admitted neonates in this study period. Thus we calculate the actual percentage of DAMA. Again all guardians seeking DAMA were interviewed to avoid selection bias.

Comment 3: Table 2 and 3 should mention p value and statistics for each variable.

Response: P value for each variable mentioned in table 2 and 3.

Comment 4: .The goodness of fit should be mentioned in statistics paragraph.

Response: Thank you for your suggestion. Now I have mentioned it in statistics section as

“To assess the accuracy of the model, a goodness-of-fit test was performed, which provided insight into how well the model fits the data.”

Comment 5: All conclusions should be based on the study results only. Terminology like medico legal etc should be supported by evidence from the study results.

Response: Thank you. The line is removed.

Comment 6: There may be many other reasons for dama or multiple causes in the same patient which should be reported and accounted for

Response: We used a questionnaire to ask about the causes of DAMA. The questionnaire was semi-structured, including a demographic profile, clinical diagnosis, and parental justifications for DAMA. The questionnaire content was developed and modified on the basis of a literature survey and focus group discussion with senior staff nurses and parents. The questionnaire was piloted among five female and five male guardians and was modified accordingly. All of the opinions collected through these steps were included in the questionnaire. Parents had to write down their choice of explanation if it differed from the ones already mentioned. So there was no chance of missing the reasons for DAMA. Only thirteen caregivers told multiple causes. Two caregivers got DAMA due to no clinical improvement of the neonate with a financial problem. Six caregivers said about the false sense of well-being with the lack of facilities for mothers/ attendants, and five caregivers were not satisfied with the treatment and mentioned the lack of facilities for mothers. As multiple responses were not included in the table, we had it under the heading of others

Following texts are added:

"Only thirteen caregivers had multiple causes to seek DAMA. Among these multiple respondents, two caregivers had financial problem and they also lost their hope for clinical improvement in the neonate. Six caregivers said about the false sense of well-being with the lack of facilities for mothers/ attendants. Five caregivers were unsatisfied with the treatment and also mentioned the lack of facilities for mothers. "

Comment 7: Discussion should be reorganized to explain the results logically.

Response: Thanks for your review. The discussion is re-written.

Comment 8: Long term outcomes of DAMA could have been reported.

Response: It was our limitation we couldn't follow-up the patient after DAMA due to financial constraints. The authors didn't get separate funding to do the work.

Comment 9: Report should follow Strobe checklist

Response: Report is written according to Strobe checklist.

Comment 10: A case control methodology would have been more sound in design of the study.

Response: Thanks for your valuable suggestion regarding case-control methodology. Following lines are added in study setting and sampling.

"This was a case-control study conducted over six month in the SCANU, Department of Neonatology, Chittagong Medical College Hospital, from July 2017 to December 2017."

"Non-probability consecutive sampling technique was used. We have enrolled the data of all neonates admitted in this study period on the day of their outcome. We defined DAMA cases as any neonate whose caregivers removed them from the hospital despite the attending physician recommending that they remain. Controls were those who were discharged on the advice of the attending physician. The number of neonatal admissions in this period was 6167. We excluded 1362 neonates who died in SCANU. Of the remaining 4805 neonates, a total of 3217 neonates were discharged with the medical advice of attending physicians. There were 1588 parents or guardians seeking DAMA. They all were included to avoid selection bias by the interviewers.

The neonates of case and control groups were matched on demography, geo-economic backgrounds or birth related variables. All controls included in this study had to meet the criteria of having minimum one individual with comparable age, sex, mode and place of delivery and diagnosis though the ratio of case: control was 1: 2.36. For every DAMA, at least two controls were available.

"

Reviewer 2

Comment 1-There needs to be more of a rationale included on the content of the questionnaire administered to caregivers.

Response: Thank you for your comment. Following lines added in the text.

"*A questionnaire was semi-structured including demographic profile, clinical diagnosis and reasons given by the parents for DAMA. The questionnaire was piloted on five female and five male guardians and was modified accordingly. The content of the questionnaire was developed and modified based on a literature search, a focus group discussion, and a pilot study. We mentioned all the opinions found from the previous steps. If parents chose a different reason other than mentioned options, they had to write it. At first, physicians and senior staff nurses*

counseled them to complete treatment, informing the prognosis and danger of discontinuation of treatment. Those intent on taking DAMA after counseling had to fill up the questionnaire where the specific cause of self-discharge had to be chosen or written by themselves or the attending senior staff nurse. The respondents usually took up to 10 minutes to complete the questionnaire."

Comment 2-Similarly, the rationale for variables assessed to compare neonates who DAMA to those who did not is needed.

Response: Thanks for raising the issue. Gestational age, birth weight, gender, place and mode of delivery, age at admission, and residence-these are the essential demographic characteristics of the neonate. Again, a previous literature search and our observation suggested that DAMA is usually increased on weekends and after office hours. Our consensus also indicated that caregivers of the neonate ask for DAMA before completing full treatment so that hospital stay may be less than the neonates who are discharged with advice. All neonates have a specific diagnosis at admission. So we compare all these variables.

Comment 3- The data only cover a six-month period that is nearly 5 years ago at this point. The rational for this time selection should be clearly explained.

Response: Admission of neonates to SCANU, Chattogram Medical College, was increased in 2017. In previous years, the number of admissions was below 9000/ year. The bed capacity and the number of physicians and senior staff nurses, and other supporting staff were the same. In this scenario, we wanted to observe the outcome of admitted neonates. We found that a significant number of neonates got DAMA. That is why we want to discover the cause of

DAMA from caregivers' perspectives. We regret the delay in publishing this article.. We discussed the findings with our hospital authority. Based on these findings, a new kangaroo-mother-care unit was established, and daily counseling by consultants with the mother and attendants was started.

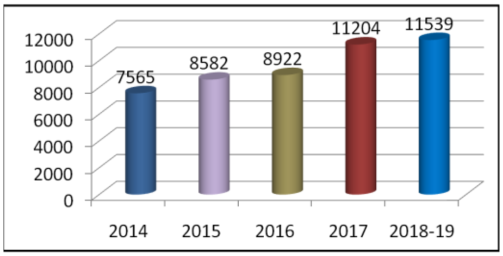

Comment 4-I think including a multivariable regression model would greatly strengthen their case.

Response: Thanks for your evaluation. I have taken the birth-related variables, demographic variables, and diagnosis of the neonates for bivariate analysis. Those which were found significant were used for logistic regression. I compare two outcomes, and the maximum variables are binary. I did not find any confounders in this study. These are the reasons I chose binary logistic regression. Multinomial logistic regression was done according to your suggestion. There is no significant variation in the result, as I could not use any confounding variables.

Comment 5-No study is perfect but the limitations paragraph gives the authors an opportunity to

acknowledge the limitations of the current study as well as call for additional work to address the limitations. I strongly suggest including one.

Response: Exactly, You are right. No study is the perfect one. I have written the limitation of my study in submitted article at the end of discussion without dividing it into different paragraph.

"*Although we included all neonates within a specific time range, this was a single center study. After DAMA, we were unable to monitor the newborns, and we lacked precise information on their prognosis. No comprehensive maternal information was recorded, which could be a contributing factor to DAMA.*"

Comment 6-Finally, I think this article should be carefully read and reviewed by a native English speaker to ensure the syntax and grammars are correct.

Response: Thanks for your suggestion. This article is carefully checked by a native English speaker.

Abstract:

Comment--Minor point, but it should be clear that the aim is to assess characteristics of neonates who DAMA and the causes and predictors of DAMA.

Response: The introduction of abstract is changed into

“The study aimed to assess the prevalence of DAMA in neonates along with characteristics of neonates who got DAMA and causes and predictors of DAMA.”

Comment--The line “A total of 6167 neonates were admitted and 1588 were self-discharged.” needs some clarification. How many were discharged against medical advice? Is that the same as “self-discharge”? I suggest being consistent with the language.

Response: The term "discharge against medical advice," "leave against medical advice," and "self-discharge" are synonymous, which I have mentioned in the introduction. The usage of the same terminology repeatedly made the readers monotonous. According to your suggestion, I have changed the line mentioned above in the abstract. Most of the “self-discharge” terminology is replaced by DAMA.

Comment--The Methods of the Abstract should include how causes were identified.

Response: The following line is added to the methods of the abstract.

“The causes of DAMA were identified by a semi-structured questionnaire.”

Comment-Does this mean urban vs rural? Needs to be clarified.

Response: It is complicated and not limited to urban and rural only. I will try to explain. Better not to clarify it in abstract. I will place it in study settings.

Chittagong Division, officially known as Chattogram Division, is geographically the largest of the eight administrative divisions of Bangladesh. No other divisions of Bangladesh have so much geographical variation like Chattogram because of the hills, the sea, the valleys and the forests. It covers the south-easternmost areas of the country, with a total area of 33,909.00 km2

Chattogram Division is presently subdivided into eleven districts (zillas) and thence into 99 sub-districts (upazilas). The first six districts (Brahmanbaria, Cumilla, Chandpur, Laskhmipur Noakhali, Feni) comprise the north-western portion (37.6%) of the division, while the remaining five (Khagrachari, Bandarban, Rangamati, Chattogram, Cox's Bazar) comprise the south-eastern portion (62.4%), the two portions being separated by the Feni River. Chattogram district is an administrative zone of the Chattogram division. Other than the Chattogram City corporation area, there are 14 subdistricts (upazilas) in Chattogram District. The upland districts of Khagrachari, Rangamati and Bandarban comprise the Chittagong Hill Tracts. Cox's Bazar is the most southern district of the Chattogram division. Sandip, Hatia, Kutubdia, Moheshkhali, and Saint Martin are the five major islands in this division we demonstrated as separate entities because of difficult access to the mainland and fewer healthcare facilities for the neonate. Thus we divide Chattogram division /residence into six classes: 1. Chattogram city corporation area, 2. Chattogram- Outside city corporation area (including 14 subdistricts/upazilas), 3. North-western districts (Brahmanbaria, Cumilla, Chandpur, Laskhmipur Noakhali, and Feni), 4. Hill Tracts (Khagrachari, Bandarban, and Rangamati), 5.Cox's Bazar, and 6.Islands (Sandip, Hatia, Kutubdia, Moheshkhali, and Saint Martin). Few lines are added in the text with a map for better understanding.

Comment--Something for the authors to consider: is a neonate who was the product of a term, vaginal delivery with standard birthweight neonate really a manifestation of a "false perception" as the authors present? I ask because those are all low-risk characteristics for untoward events in the neonatal period.

Response: Thanks for raising the issue. In our study, caregivers of 370 term neonates had a false perception of well-being. About 274 neonates weighed more than 2500 grams in this group. Our

traditional belief is that term, normal birth weight babies are the healthier ones. But these neonates were admitted here with specific diagnoses. I agree that some babies may be planned for early discharge. Mostly, perinatal asphyxia (with or without hypoxic-ischemic encephalopathy stage 1) without other complications might be discharged earlier. But caregivers didn't wait for that. Many of them were discharged within 24 hours. But a good number of term and normal birth weight neonates with perinatal asphyxia with hypoxic-ischemic encephalopathy stage 2 and stage 3, sepsis (early and late onset) got DAMA. Lip smacking and cycling may be the manifestation of seizures that the parents couldn't recognize as disease manifestations. Again we couldn't discharge them without full assessment and without fulfilling the discharge criteria. It may be the cause of false perception.

Comment--The odds ratio for "timing of outcome after office hours" is gigantic. Is this true? I don't think I have ever seen an odds ratio of that magnitude.

Response: You have correctly picked up the problem. It's really gigantic. Most of the discharge with advice was planned in the morning within office hours. On the other hand, DAMA happened throughout the morning, evening, and night. This may be the cause for the gigantic odds ratio.

Comment--The sentence "Diagnosis and residence also played roles in their decision making" is vague and needs to be reworded. Which direction was the relationship?

Response: Thanks. Following lines are added.

"Neonates suffering from sepsis (OR 1.4, 95% CI 1.1-1.7, p<0.001), Respiratory Distress Syndrome (OR 3.1, 95% CI 1.9-5.2, p <0.001), prematurity without other complications (OR 2.1, 95% CI 1.45-3.1, p <0.001) or who were referred from north-western districts (OR 1.48, 95% CI 1.13-1.95, p=0.004) had higher odds for DAMA."

Introduction:

Comment--The sentence "So, treating doctors still worry about getting into trouble with the law." is unclear. Are doctors worried about being sued when treating neonates any more so than treating other patient populations? If so, why?

Response: Thanks for your suggestion. For clarification, we removed the sentence. No doctors were sued for DAMA in our institution. Doctors are mainly worried about patient outcomes. Another issue is that DAMA is an indicator of hospital services, and we have to enter the percentage of DAMA on the government website. The hospital authority made a query for an increased rate of DAMA.

Comment-Again, why use "self-discharge" and "DAMA" throughout the paper? It is best to be consistent.

Response: The term "discharge against medical advice," "leave against medical advice," and "self-discharge" are synonymous, which I have mentioned in the introduction. The usage of the same terminology repeatedly made the readers monotonous. I have changed the line mentioned above in the abstract. Most of the "self-discharge" terminology is replaced by DAMA.

Methods:

Comment--More of a description of the study setting would be helpful to the reader to see how these results do, or do not, compare to settings where they may work.

Response: Detailed description of study setting has been added.

Comment-By the description, it seems that many of the neonates were term, normal birthweight, etc., which makes me wonder why they would need much care at all.

Response: Our traditional belief is that term, normal birth weight babies are the healthier ones. But these neonates were admitted here with specific diagnoses. I agree that some babies may be planned for early discharge. Mostly, perinatal asphyxia (with or without hypoxic-ischemic encephalopathy stage 1) without other complications might be discharged earlier. But caregivers didn't wait for that. Many of them were discharged within 24 hours. But a good number of term and normal birth weight neonates with perinatal asphyxia with hypoxic-ischemic encephalopathy stage 2 and stage 3, sepsis (early and late onset) got DAMA. Lip smacking and cycling may be the manifestation of seizures that the parents couldn't recognize as disease manifestations. Again we couldn't discharge them without full assessment and without fulfilling the discharge criteria. It may be the cause of false perception.

-In order to demonstrate rigor, the content of the questionnaire administered to caregivers needs some basis of the content. I applaud the authors for piloting it with 10 caregivers, but where did the content come from? Ideally, it should be a validated tool, but in the absence of such, pointing at the results of prior studies may suffice.

Response: Thanks for your appraisal. This was not a validated tool. The questionnaire was semi-structured, including demographic profile, clinical diagnosis, and reasons given by the parents for DAMA. The questionnaire's content was developed and modified on the basis of a literature survey and focus group discussion (FGD) with senior staff nurses and parents. The questionnaire was piloted among five female and five male guardians and was modified accordingly. We mentioned all the opinions in the questionnaire found from these steps. If parents chose a different reason other than mentioned options, they had to write it. Finally, the respondents took up to 10 minutes to complete the questionnaire.

Comment-It appears to be quantitative but again, the rationale for the content and how the questions were posed is needed.

Response: Yes, It is quantitative. The rationale for the content and how the questions were posed is described in the previous response.

Comment-Yes, bivariate comparisons are nice, but it is stronger to show which factors are independently associated with the outcome adjusting for potential confounders whenever possible.

Response: Thanks for your evaluation. I have taken the birth-related variables, demographic variables, and diagnosis of the neonates for univariate analysis. Those which were found significant were used for logistic regression. I compare two outcomes, and the maximum variables are binary. I did not find any confounders in this study. These are the reasons I chose binary logistic regression. Multinomial logistic regression was done according to your suggestion. There is no significant variation in the data, as I could not use any confounding

variables.

Results:

Comment-Referring to places like “Cumilla” and “Cox’s bazar” is not helpful for the reader as it implies they know where these are. I suggest describing these settings instead. Are they urban, rural, etc.?

Response: Thanks for your valuable suggestion. The name of different districts is explained on the basis of distance and geographical characteristics. Following lines are added in the study setting.

“Chattogram Division is presently subdivided into eleven districts (zillas) and thence into 99 sub-districts (upazilas). The first six districts (Brahmanbaria, Cumilla, Chandpur, Laskhmipur Noakhali, and Feni) comprise the division's north-western portion (37.6%). In contrast, the remaining five (Khagrachari, Bandarban, Rangamati, Chattogram, Cox's Bazar) comprise the south-eastern portion (62.4%), the two portions being separated by the Feni River. Chattogram district, an administrative zone of the Chattogram division, has 14 subdistricts (upazilas) apart from Chattogram city corporation area. The upland districts of Khagrachari, Rangamati, and Bandarban comprise the Chittagong Hill Tracts. Cox's Bazar is the most southern district of the Chattogram division. There are different islands in this division: Sandip, Hatia, Kutubdia, Moheshkhali, and Saint Martin. Islands have difficult access to the mainland and fewer healthcare facilities for the neonate. Neonates from the entire division are referred to this SCANU, which covers a total area of 33,909.00 km2.”

Comment-The comparison made by “length of stay needs for DAMA vs not” should be by median length of stay, not chi square. I suggest this because the description of “term, vaginal delivery with standard birthweight neonate” among those who DAMA is descriptive of a healthy population. So it makes me wonder if they needed to be in the hospital longer than a day, or why such a neonate is admitted to the SCANU.

Response: The data of hospital stay of individual neonate are not parametric. Mean hospital stay is not applicable to nonparametric data. This is the reason; we changed hospital stay into duration of hospital stay.

Term (n=3933) with average birth weight babies were admitted with specific disease entity. About 1176 term babies got DAMA. Among them 160 patients were diagnosed as perinatal asphyxia with HIE stage 1. They were planned to discharge after 24-48 hours. Most of them (84) didn’t wait even for 24 hours. But other neonates got DAMA within 24 hours were sick and not meet the criteria of discharge.

Comment-The “Result (Chi square)” probably isn’t necessary to include. I don’t think I have seen this in any paper. The p value for a Chi square test is sufficient.

Response: Thanks for suggestion. Chi square values are removed.

Comment-Table 2: Please spell out all abbreviated diagnoses for clarity.

Response: Elaborated diagnoses are used in the text and in Table 2 to improve clarity.

Comment-Table 2: I defer to the editor and a biostatistical reviewer, but a single Chi square for this entire table is misleading. I suggest comparisons be made by each row instead.

Response: Thank you suggesting p value for each row. Table 2 is updated accordingly.

Comment-Why is the “percentage of DAMA estimated as 25.7%”? That seems like a precise proportion and not an estimate.

Response: The word ‘estimated” is replaced. The line is changed as

The percentage of DAMA was calculated at 25.7% in this work.

Comment-Table 3: Please clarify in the title that these were ascertained from caregivers.

Response: The title of Table 3 is changed as

“Cause behind DAMA according to care-givers’ response”

Comment-Table 3: Again, I think row by row comparisons are more meaningful than one for the entire table.

Response: Thank you suggesting p value for each row. Table 3 is updated accordingly.

Comment-Table 4 does not need the beta coefficients or standard errors. Odds ratios, 95% CIs +/- P values should suffice.

Response: Column mentioning beta coefficients and standard errors were removed from Table 4.

Discussion:

-Is DAMA a “cause of patient dissatisfaction” or a manifestation of it? I suspect it is the latter.

Response: Yes. I agree with your opinion. DAMA is the manifestation of patient dissatisfaction.

-The first part of paragraph 2 would be better suited in a description of the Study Setting in a section in the Methods.

Response: Study setting and demographic characteristics of the division is described.

-In general, the Discussion reads as a bit rambling and could be focused. I suggest the authors spend time thinking more about the implications of each finding and trying to tie that into each paragraph.

Response: Thanks for your review. Discussion is re-written.

-Is the final paragraph a Conclusions paragraph? If so, I suggest clearly labeling it as such.

Response: Yes. Final paragraph is the conclusion. I thought that conclusion is optional. Now conclusion is clearly mentioned. Thank you for your suggestion.

Reviewer 3:

Comment 1: Main finding of the paper Study period-6 months (from July-December 2017)

• Bed occupancy rate -141 %(unacceptably high)

• One thousand five hundred eighty (25.7%) of the patients discharge against medical advice (DAMA) among 6167 admitted neonate in the study period the prevalence is very high.

• Main reason for DAMA were false perception of well-being of neonates, inadequate facility for mothers and financial reason

• Main predictors for DAMA were preterm delivery, vaginal delivery, and timing of outcome after office hours

No major gaps identified in abstract, introduction and methods section)

Response: Thank you for your intense observation. You correctly picked up the main findings. As the DAMA prevalence was high, we wanted to find out the causes.

Result section:

Comment: • The authors used old data set (2017)

I would like to thank you very much to reviewer 1 for reviewing the manuscript and providing your expert comments and opinions on the manuscript. All the comments were welcomed and carefully studied. We apologize for our late publication. Actually we discussed the study findings with our hospital authority. On the basis of these findings, a new kangaroo-mother-care unit was established and daily counseling by consultants with the mother and attendants were

started. It is expected that this sort of study will always help to undertake similar relevant steps for better patient management and outcome.

Comment: The description of the result is not coherent and the sentences are fragmented it is not eye catching (need rewriting)

Response: The description of the result is re-written and checked by a native English speaker.

Comment: Better to put the main finding of tables in summarized ways (as long as you annexed the table within the paragraph)

Response: Thank you for the suggestion. I have tried to explain the main findings of the table in summarized way.

Comment: While summarizing the result, mentioning percentage (%) alone may not give sense. so it is better to report as Number (%) uniformly.

Response: Number is added along with the percentage wherever applicable.
