# Supplementary material for: Discharge against medical advice in Special Care Newborn Unit in Chattogram, Bangladesh: Prevalence, causes and predictors
Source: PLoS One. 2023 Apr 20;18(4):e0284705. doi: 10.1371/journal.pone.0284705 (PMC10118186; doi:10.1371/journal.pone.0284705)
Supplement: Attachment — Submitted filename: Rebuttal letter.2.doc [file pone.0284705.s003.doc]

**Detailed response to reviewers:**

Comment 1: During your initial submission of your manuscript, you mentioned that the study design was cross-sectional study design. However, in the revised manuscript, you have stated that "case-control" study design was used. How the change in the study design was made during manuscript write up stage is not clear. Please clarify it.

Response: Thank you for your question. We enrolled every neonate admitted from July 2017 to December 2017 on the day of their outcome. Initially, we planned for a cross-sectional study design during protocol submission because the number of neonates in each group (Discharge with advice, DAMA, and Death) was expected to be different. However, after data collection, we excluded the deaths. Among the remaining two groups, most of the neonates were matched on demographic, geo-economic backgrounds, or birth-related variables. The ratio of case to control was 1:2.36, with at least two controls available for every DAMA case. We wanted to identify the predictors of DAMA, so we decided that the case-control methodology was more appropriate. We had mentioned the study as a cross-sectional analytical study in our protocol and initial submission. Reviewer#1 suggested that case-control methodology would be more sound. After receiving feedback from the reviewer#1 and considering our analysis, we realized that a case-control study design would be more appropriate to achieve the research objectives. Therefore, we revised the manuscript to reflect the change in study design. We have already addressed this concern in our previous response but are happy to provide additional clarification if needed. We apologize for any confusion caused by this change.

Comment 2: Please remove map of Bangladesh from the manuscript. It doesn't have direct relationship with your research findings.

Response: Thanks for your suggestion. Map of Bangladesh is removed from the revised manuscript.

Comment 3: Your description of the study setting has to be concise.

Response: Thank you for your suggestion. The study setting has been concise as follows:

“*This case-control study was conducted over a period of six month from July 2017 to December 2017 at the SCANU in Department of Neonatology, Chittagong Medical College Hospital. It is one of the largest SCANUs in the country, with 32 government allocated beds and 56 locally arranged beds. The average number of admitted neonates ranges from 160-210 per day in 100 cots. Admission criteria are gestational age less than 34 weeks, birth weight less than 1800 g, and any sick neonate irrespective of gestational age and birth weight. Neonates from the entire division are referred to this SCANU, which covers a total area of 33,909.00 km2. As a tertiary and referral hospital, admission of sick neonates can not be denied in the SCANU.As a result, more than one neonate share the same cot. During study period, five consultants including two professors were working in the SCANU. There were 8–10 mid-level doctors who were on rotation from pediatrics as part of their neonatal training, and approximately 40 senior staff nurses were working in three shifts.*

*The Chattogram division, located in Bangladesh, consists of 11 districts and 99 sub-districts.(16) Within this division, Chattogram district serves as the administrative zone. In this study, the division was classified into six areas based on administrative zone, geographic characteristics, and access to healthcare facilities for neonates. These are Chattogram district urban (city corporation area), Chattogram district non-urban (14 sub-districts apart from city corporation area), North-western districts (6 districts), Hill tracts (3 upland districts), Cox’s Bazar (1 coastal district) and Islands.”*

Comment 4: In your findings of Binary Logistic Regression, please use Adjusted Odds Ratio (AOR) not the crude Odds Ratio.

Response: Thank you for your comment regarding the odds ratio reported in our analysis. A multivariate binary logistic regression analysis was conducted in our study including all significant variables identified in the univariate analysis. We apologize for the confusion caused by our labeling the odds ratio as crude when it was actually adjusted. We understand that the crude odds ratio is an unadjusted estimate of the association between the predictor and outcome variables, while the adjusted odds ratio is a more precise estimate that takes into account the effects of other variables in the model. We revised our manuscript to ensure that we use the correct terminology in the manuscript.
